# Diagnostic Biomarkers to Diagnose Acute Allograft Rejection After Liver Transplantation: Systematic Review and Meta-Analysis of Diagnostic Accuracy Studies

**DOI:** 10.3389/fimmu.2019.00758

**Published:** 2019-04-11

**Authors:** Felix Krenzien, Eriselda Keshi, Katrin Splith, Silvan Griesel, Kaan Kamali, Igor M. Sauer, Linda Feldbrügge, Johann Pratschke, Annekatrin Leder, Moritz Schmelzle

**Affiliations:** ^1^Department of Surgery, Charité-Universitätsmedizin Berlin, Campus Charité Mitte and Campus Virchow-Klinikum, Berlin, Germany; ^2^Berlin Institute of Health Research, Berlin, Germany

**Keywords:** liver transplantation, acute rejection, diagnostic biomarker, diagnostic accuracy, non-invasive test

## Abstract

**Objective:** A systematic review and meta-analysis of diagnostic biomarkers for noninvasive diagnosis of acute allograft rejection following liver transplantation.

**Background:** Noninvasive blood and urine markers have been widely explored in recent decades for diagnosing acute rejection after liver transplantation. However, none have been translated into routine clinical use so far due to uncertain diagnostic accuracy, and liver biopsy remains the gold standard.

**Methods:** Systematic literature searches of Medline, Cochrane and Embase were conducted up to February 2019 to identify studies evaluating the use of noninvasive markers in diagnosing allograft rejection following liver transplantation. Meta-analysis was performed using a random effects model with DerSimonian–Laird weighting and the hierarchical summary receiver operating curve.

**Results:** Of 560 identified studies, 15 studies (1,445 patients) met the inclusion criteria. The following markers were tested: acid labile nitroso-compounds (NOx), serum amyloid A protein, procalcitonin, peripheral blood eosinophil count, peripheral blood T-cell activation and interleukin 2 (IL-2) receptor, guanylate-binding protein-2 mRNA, graft-derived cell-free DNA, pi-glutathione S-transferase, alpha-glutathione S-transferase and serum HLA class I soluble antigens. Only eosinophil count was tested in multiple studies, and they demonstrated high heterogeneity (*I*^2^ = 72% [95% CI: 0.5–0.99]). IL-2 receptor demonstrated the highest sensitivity (89% [95% CI: 0.78–0.96]) and specificity (81% [95% CI: 0.69–0.89]).

**Conclusion:** IL-2 receptor expression demonstrated the highest diagnostic accuracy, while the peripheral eosinophil count was the only marker tested in more than one study. Presently, liver biopsy remains superior to noninvasive diagnostic biomarkers as most studies exhibited inferior designs, hindering possible translation into clinical application.

## Introduction

Liver transplantation (LT) exhibits 20-year survival rates of up to 50% (1) and is of great clinical importance. LT is the treatment of choice for acute or acute-on-chronic liver failure, while organ replacement therapies are still waiting for the break (2). LT is superior to liver resection in suitable patients with hepatocellular carcinoma in cirrhosis, the most common primary liver cancer and the second leading cause of cancer-related deaths worldwide (3, 4).

Within the first few weeks after transplantation, patients are at high risk of acute rejection (AR), with the incidence ranging from 50 to 70% (5), depending on the immunosuppressive regime selected. AR can be described as an immune response against donor tissues resulting from T-cell recognition of allo-antigens. This overwhelming immune response compromises graft integrity and can lead to life-threatening graft loss. Thus, AR is the most common cause of transplant failure and the most common indication for re-transplantation. Indeed, the early diagnosis of AR is crucial for successful anti-rejection therapy and maintenance of graft function/integrity. The importance of prompt AR diagnosis and management is increased due to an organ shortage and an increasing proportion of marginal organs.

The liver graft and liver function can be monitored by standard blood tests such as total bilirubin, alanine aminotransferase, aspartate aminotransferase, γ-glutamyl transpeptidase, and alkaline phosphatase. Leukocytosis and eosinophilia are also frequently present (6). In addition, the trough blood levels of immunosuppressive drugs can be monitored and may predict AR risk (7). Nevertheless, standard laboratory tests are nonspecific and are not suitable for the efficient and timely diagnosis of AR (8). In the case of suspected AR, liver biopsy with histologic specimen diagnosis and grading remains the diagnostic tool of choice in routine clinical practice. Nonetheless, liver biopsy is an invasive procedure associated with severe complications primarily performed by trained colleagues in transplant centers.

The concept of noninvasive measurement applies to biomarkers in the saliva, peripheral blood, urine or other body fluids (e.g., cytokines or surface proteins of different immune cells) (8). Such diagnostic biomarkers have been explored to replace liver biopsy and are predominantly used in the field for diagnostic and monitoring purposes. The first attempt to noninvasively diagnose allograft rejection in LT patients occurred 30 years ago (9). Since then, numerous studies have been published, but a single method has not yet been adopted into routine clinical use. New biomarkers are compared with liver biopsy in terms of their usefulness for diagnosing AR.

Markers for AR have to face sensitivity and specificity, statistical measures that determine the applicability of diagnostic tests. Sensitivity, the true positive rate, refers to how well a test identifies transplant patients who are suffering from AR. Specificity is the true negative rate and is of particular interest because most known markers are not able to discriminate AR from liver dysfunction, cytomegalovirus infection and hepatitis C virus infections (8). Indeed, a diagnostic tool must be validated for accuracy, otherwise its significance remains uncertain. In addition, the test should be easily performed with results that are available on the same day so that anti-rejection treatment may be initiated as soon as possible.

Therefore, the aim of this study was to perform a systematic review and meta-analysis evaluating the diagnostic accuracy of noninvasive markers in the diagnosis of AR compared with conventional liver biopsy in patients following LT.

## Methods

The systematic review was conducted according to the Preferred Reporting Items for Systematic Reviews (PRISMA) (10) guidelines and was registered with the International Prospective Register of Systematic Reviews (PROPSPERO: CRD42017072425).

### Literature Search

The author searched MEDLINE (via PubMed), EMBASE and the Cochrane Library electronic databases. No date restriction was applied, and the literature search was performed in August 2017 and updated in February 2019. The search terms were initially reviewed by the author group and were sent to an expert librarian to ensure completeness and accuracy according to the PRESS Guideline Statement (11). The specific search strategy for MEDLINE/PubMed conducted in January 2018 was: Search ((((((((((((((“acute rejection”) OR (“acute allograft rejection”) OR (“acute graft rejection”) OR (“acute liver allograft rejection”) OR (“acute reject^*^”) OR (“acute cellular rejection”) OR (ACR) OR (“early rejection”) OR (“early reject^*^”) OR (“early allograft rejection”) OR (“early graft rejection”) OR (“early liver allograft rejection”) OR (“early liver graft rejection”) OR (“early cellular rejection”)))) AND ((biomarker OR biomarkers OR marker OR non-invasive OR liquid-biopsy))) AND (((liver transplantation) OR (liver graft^*^) OR (liver transplant^*^)))) AND (((sensitiv^*^[Title/Abstract] OR sensitivity and specificity[MeSH Terms] OR (likelihood ratio) OR odds ratio OR (OR) OR (predictive value) OR AUC OR AUROC OR (area under curve) OR LR OR diagnose[Title/Abstract] OR diagnosed[Title/Abstract] OR diagnoses[Title/Abstract] OR diagnosing[Title/Abstract] OR diagnosis[Title/Abstract] OR diagnostic[Title/Abstract] OR diagnosis[MeSH:noexp] OR diagnostic ^*^[MeSH:noexp] OR diagnosis, differential[MeSH:noexp] OR diagnosis[Subheading:noexp]))))))))))). The same search strategy was applied for the Cochrane Library and EMBASE as well.

#### Selection Criteria

Studies were included in the systematic review if they met the following criteria:

- The study was designed as a diagnostic accuracy trial testing a noninvasive biomarker(s) of AR in LT patients.
- AR was defined according to the International Consensus Document on Terminology of Hepatic Allograft Rejection (6).
- The index test was primarily used to diagnose AR.
- The study used liver biopsy and histopathological grading as the reference test to diagnose AR.
- The study presented sufficient data to create a 2 × 2 contingency table.
- The study enrolled adult patients (≥18 years of age).
- The study was published in English.

Prediction of AR at some time in the future or the grading of patient risk for AR at any time point following LT led to study exclusion, because predicting the likelihood of AR in the future is different than diagnosing AR in clinical practice where anti-rejection therapy must be initiated on the same day as diagnosis. Therefore, studies needed to specify the time interval between sampling and confirmation of BPAR (biopsy-proven acute rejection), ensuring that test samples were timely connected to BPAR for analysis.

### Study Selection and Data Extraction

All articles identified by the search were screened and excluded if they did not meet the inclusion criteria. The full texts of all potentially relevant studies were reviewed in detail. Data was extracted using a predetermined standardized form and included the following information: study design (cohort/single-gate or case-control/multi-gate), characteristics of participants, time of follow-up and regimen of immunosuppressive therapy. Test validity was assessed by the total number of patients with AR/no AR, prevalence of AR in the sample, and test parameters including sensitivity, specificity, positive predictive value (PPV), negative predictive value (NPV), likelihood ratio (LR), and area under the curve (AUC).

### Risk of Bias Assessment

The Revised Quality Assessment of Diagnostic Accuracy Studies tool (QUADAS-2) was used to appraise the reliability and applicability of the study findings (Supplementary Table 1) (12). The signaling questions were carried out independently by two reviewers (FK and EK). Any discrepancy between the reviewers was resolved through discussion until a common conclusion was achieved. The assessment tested for patient selection, index test, reference standard, and study flow and timing. Each item was answered by *yes, no* or *unclear*, indicating a high, low or unclear risk of bias.

#### Statistical Analysis

Contingency tables were constructed based on values for sensitivity, specificity and corresponding sample size given in the studies. The extracted data was then used to calculate the PPV, NPV, and the positive and negative LR for each test when not reported in the manuscript. Confidence intervals were demonstrated with forest plots (13).

After critical appraisal, a meta-analysis was conducted to pool study estimates of specific markers. All pooled outcome measures were determined using the random effect model described by DerSimonian and Laird. The risk ratio (RR) of patients positive for eosinophilia against the risk of patients negative for eosinophilia to suffer from AR after transplantation confirmed by liver biopsy was estimated for each study. Heterogeneity among studies was quantified using the *I*^2^ statistic, which describes the percentage of variation across studies due to heterogeneity rather than chance (14). The RR of each study was plotted against the respective measures of study size to investigate any existing bias (15) and was visualized through a funnel plot. Lastly, a hierarchical summary receiver operating curve (HSROC), as described by Rutter and Gatsonis, was used to simultaneously estimate the summary receiver operating curve (SROC) and the expected operating point on the curve for the diagnostic accuracy studies testing eosinophilia (16). All statistical analyses were conducted using statistical software R (www.r-project.org), and *P* < 0.05 was considered significant.

## Results

**Figure 1:**
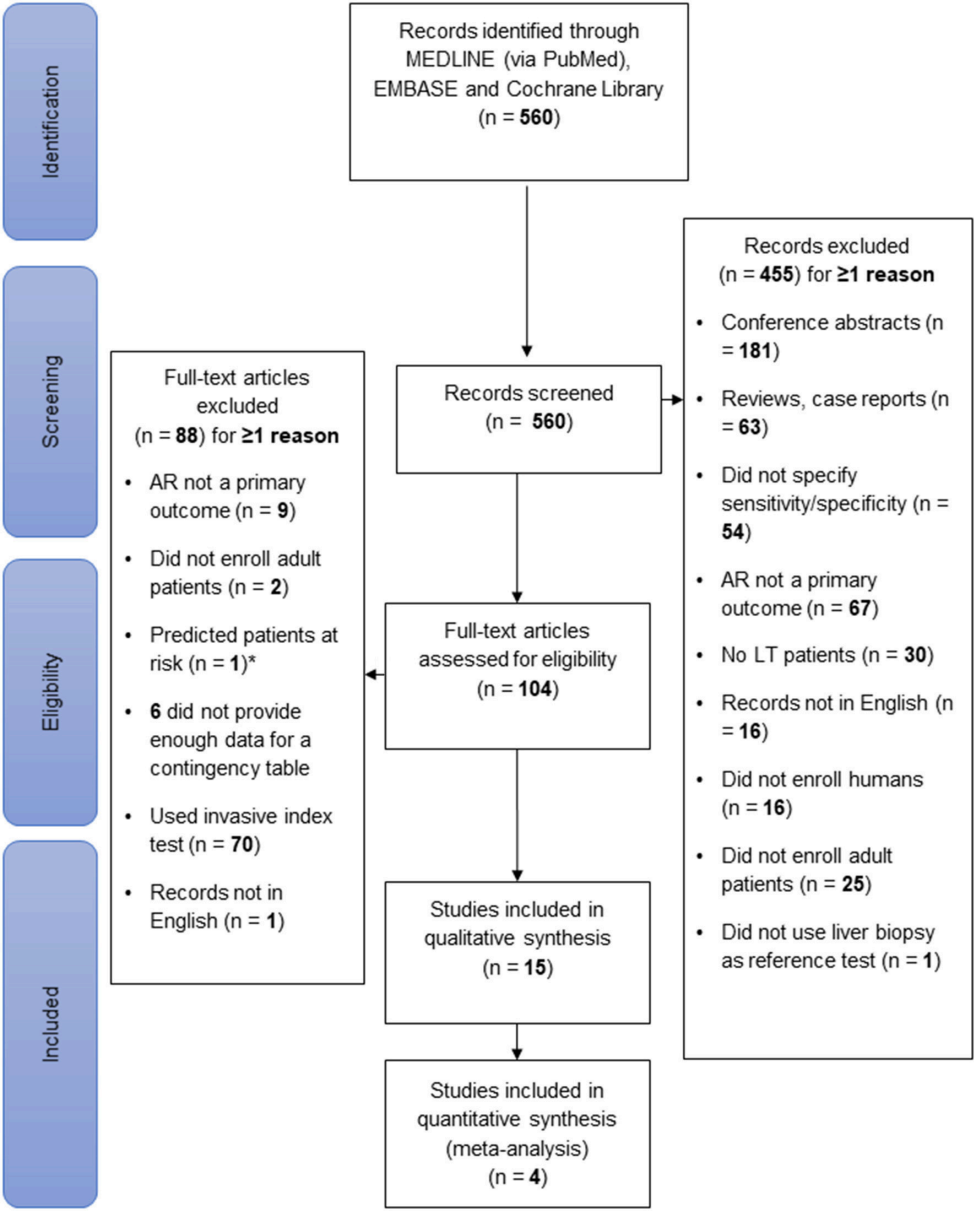
Flow of information during study selection. The literature search was conducted in Medline, the Cochrane Library and Embase in February 2019. Of 560 identified records, 15 were selected for systematic review. Of the 104 full-text articles assessed for eligibility, 88 were excluded. Of note, only predicting the risk of AR development, not diagnosing AR, was considered an exclusion criterion. This figure was designed according to the PRISMA-Statement (10).

Of the initial 560 references retrieved from the databases, 104 were filtered for full-text review after the titles and abstracts were screened. Of these, only 15 studies fulfilled all inclusion criteria and were included in the systematic review. A PRISMA flow chart depicting the flow of information through the different phases of the literature review is shown in Figure 1. All studies were published between 1994 and 2019.

### Baseline Study Characteristics

The lead author's name, study center, study design, studied index test, follow-up period and sample size are listed for each included study in Table 1. A risk of bias assessment using the QUADAS-2 tool was carried out for all studies (12), and only 6 studies exhibited low bias in all four parameters (Table 2). The overall allocation of risk of bias of the included studies is graphically depicted in Supplementary Figure 2. In total, 12 studies were prospective consecutive cohort trials, 2 studies identified markers by exploration and validation, and 1 study was retrospective in design. Overall, 1,446 patients were analyzed by the included studies, and all patients underwent LT due to different underlying liver diseases.

**Table 1:** Characteristics of diagnostic studies.

**Author**	**Center**	**Design**	**Index test**	**Sample size**	**Acute rejection (*n*)**	**Follow-up**
Devlin et al. (17)	Institute of Liver Studies, Kings College School of Medicine, London, UK	Prospective cohort trial (consecutive)	NOx (acid labile nitroso compounds)	50/50 patients included Test samples = 55	33	28 days
Feussner et al. (18)	Universität Heidelberg, Abteilung Innere Medizin, Endokrinologieund Stoffwechsel, Germany	Prospective cohort trial (consecutive)	Serum Amyloid A protein	12/12 patients included Test samples = 42	14	70 days
Kuse et al. (19)	Medizinische Hochschule Hannover, Viszeral und Transplantationschirurgie, Hannover, Germany	Open prospective cohort trial (consecutive)	Procalcitonin	20/40 patients included; Test samples = 40	10	2 weeks
Okubo et al. (20)	Graduate School of Medicine and Immunology Frontier Research Center, Osaka University, Suita, Osaka, Japan	Exploratory study	CHMP2B KCTD14 KCNAB3 TPI1	80/80 patients included Test samples = 80	20	1 year
Hughes et al. (21)	Department of Clinical Biochemistry, Addenbrooke's Hospital, Cambridge, England	Prospective cohort trial (consecutive)	EOS (eosinophil count), ECP (eosinophil cationic protein)	51/51 patients included Test samples = 71	36	100 days
Lun et al. (22)	Insitut für Laboratoriumsmedizin und Pathobiochemie, Campus Virchow Klinikum,Berlin, Germany	Prospective cohort trial (consecutive)	Peripheral blood T-Cell activation and IL-2 Receptor	119/119 patients included Tests samples = 119	69	20 days
Barnes et al. (23)	Liver Transplant Unit, Royal Free Hospital, Pond Street, London, UK	Consecutive cohort study	Blood eosinophilia	101/101 patients included Test samples = 275	166	2 weeks
Kobayashi et al. (5)	Department of Surgery, Osaka University, Suita, Osaka 565-0571, Japan	Prospective cohort trial	Guanylate-binding protein 2 mRNA	46/46 patients included Test samples = 46	19	Unclear
Massoud et al. (24)	Division of Gastroenterology and Hepatology, University of Alabama at Birmingham, Birmingham, AL, USA	Exploratory study	Proteomics and ELISA (C4)	62/62 patients included Test samples = 62	33	7 days
Rodriguez-Peralvares et al. (25)	The Royal Free Sheila Sherlock Liver Centre and University Department of Surgery, Royal Free Hospital London, UK	Prospective cohort trial	Blood eosinophil count	615/690 patients included Test samples = 690	532	14 days
Wang et al. (26)	Liver Transplantation Center, the Third Affiliated Hospital, Sun Yat-Sen University, Guangzhou, China	Retrospective cohort trial	Blood eosinophil counts	37/37 patients included Test samples = 40	24	6 months
Schütz et al. (27)	Clinical University Hospital, “Virgen Arrixaca”-IMIB, Murcia, Spain.	Prospective observational multicenter cohort trial	Graft-derived cell-free DNA	115/115 patients included Test samples = 107	107	1 year
Dickson et al. (28)	Section of Hepatobiliary Diseases, University of Florida, Gainesville, USA.	Prospective cohort trial	Alpha-GST and Pi-GST	44/52 patients included Test samples = 44	14	7 days
Nagral et al. (29)	Hepatobiliary Medicine and Liver Transplantation, Royal Free Hospital School of Medicine, London, United Kingdom	Prospective cohort trial (consecutive)	Plasma alpha-glutathione S transferase	23/23 patients included Test samples = 56	38	46 days
Renna Molajoni et al. (30)	Divisione Tranpianti Dórgano,Catedra di Patologia, Chirurgica II, La Sapienza University, Rome, Italy	Prospective cohort trial (consecutive)	Serum HLA class I antigen	14/14 patients included Test samples = 16	8	30 days

*Systematic literature searches (Medline, Cochrane Library, and Embase) were conducted to identify studies that evaluated biomarkers to diagnose allograft rejection in patients following liver transplantation. Studies were included when the non-invasive index test(s) and reference test (liver biopsy) were performed at the same time and the sensitivity and specificity were given (n = 15)*.

**Table 2:** Assessing risk of bias and concerns regarding the applicability of diagnostic studies.

**Study**	**Risk of bias**				**Concerns regarding applicability**		
	**Patient selection**	**Index test**	**Reference standard**	**Flow and timing**	**Patient selection**	**Index test**	**Reference standard**
Nagral et al. (29)	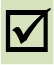	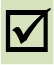	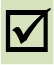	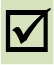	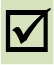	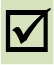	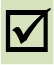
Devlin et al. (17)	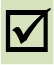	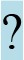	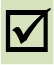	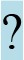	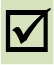	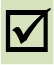	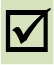
Feussner et al. (18)	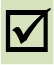	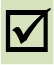	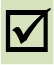	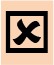	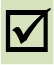	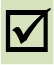	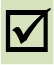
Kuse et al. (19)	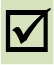	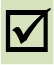	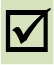	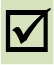	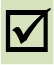	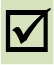	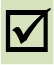
Okubo et al. (20)	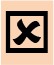	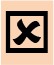	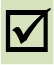	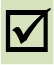	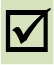	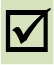	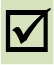
Hughes et al. (21)	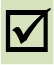	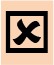	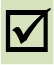	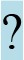	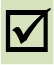	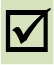	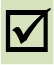
Lun et al. (22)	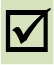	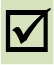	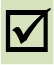	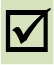	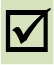	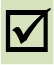	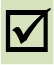
Barnes et al. (23)	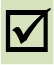	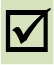	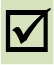	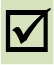	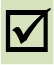	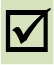	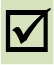
Kobayashi et al. (5)	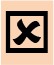	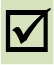	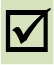	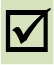	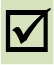	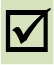	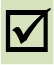
Massoud et al. (24)	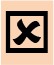	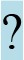	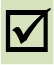	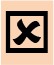	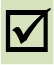	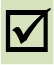	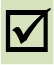
Rodriguez-Peralvarez et al. (25)	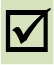	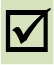	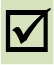	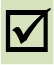	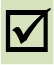	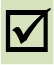	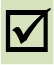
Wang et al. (26)	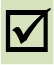	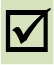	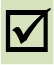	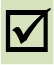	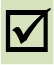	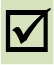	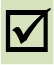
Schütz et al. (27)	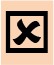	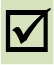	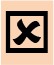	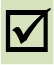	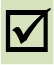	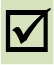	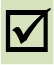
Dickson et al. (28)	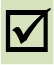	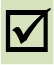	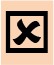	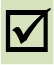	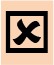	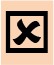	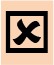
Molajoni et al. (30)	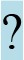	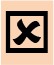	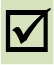	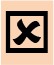	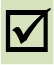	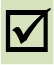	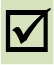

*The Quality Assessment of Diagnostic Accuracy Studies tool (QUADAS-2) was used to appraise trustworthiness and applicability of the study findings (12). The signaling questions were carried out independently by two reviewers (F.K. and E.K.). Any discrepancy between the reviewers was resolved through discussion until a common conclusion was achieved. The analysis found that Barnes et al. (23), Nagral et al. (29), Kuse et al. (19), Lun et al. (22), Rodriguez-Peralvarez et al. (25), and Wang et al. (26) exhibited low risk*.

*
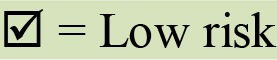

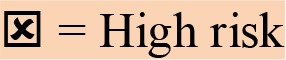

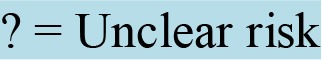
*.

Index tests were measured by taking peripheral blood samples at specific pre- or post-transplant time points, and liver biopsies were performed timely connected to the index tests. Note, liver biopsy was used as the reference standard in all included studies (*n* = 15). The markers identified by our review were eosinophilia (4 studies, 805 patients), serum amyloid A protein (1 study, 12 patients), nitric oxide (1 study, 50 patients), alpha-glutathione S-transferase (2 studies, 67 patients), pi-glutathione S-transferase (1 study, 44 patients), peripheral T-cells and soluble IL-2 receptor (1 study, 119 patients), guanylate-binding protein 2 mRNA (1 study, 46 patients), graft-derived cell-free DNA (1 study, 115 patients), procalcitonin (1 study, 20 patients), and serum HLA class I soluble antigens (1 study, 14 patients). Serum proteome characterization and subsequent validation through ELISA were performed in two studies. The study by Massoud et al. (24) identified the following seven markers: serum amyloid A protein (SAA), complement 4 (C4), fibrinogen, complement 1q (C1q), complement 3, heat shock protein 60, and heat shock protein 70. The study by Okubo et al. (20) identified autoantibodies in sera by fold change and intensity to charge one of the following markers: multivesicular body protein 2B, potassium channel tetramerization domain containing 14, voltage-gated subfamily A regulatory beta subunit 3, and triosephosphate isomerase 1.

The primary assessed outcome was index test accuracy (sensitivity, specificity, PPV, NPV, +LR, and –LR) in AR diagnosis confirmed by liver biopsy. The index test was usually performed on the same day or one day before liver biopsy. In 7 studies, the diagnostic accuracy was tested by the combination of the index test and an additional liver function parameter.

### Noninvasive Marker Test Accuracy and Meta-Analysis

All test parameters are listed in Table 3. When insufficient data were provided in the studies, test parameters were calculated using a conventional contingency table. Out of all the parameters explored by the 15 included studies, soluble IL2-R, studied by Lun et al. (22), demonstrated superior accuracy; in 119 patients a threshold value of >631 IU/ml predicted AR with a high sensitivity (81%) and a specificity of 89%. In addition, the study was assessed as having a very low risk of bias according to the QUADAS-2 tool and had a considerable sample size (Table 2).

**Table 3:** Test accuracy of diagnostic studies.

**Author**	**Index test**	**Cut-off**	**Number of patients**					**PPV (%)**	**NPV (%)**	**+LR**	**–LR**	**Sensitivity (95% CI)**	**Specificity (95% CI)**	**Sensitivity**	**Specificity**
			**TP**	**FP**	**TN**	**FN**	**Accuracy (%)**								
Barnes et al. (23)	Eosinophilia	Unclear	**53**	**12**	**97**	**113**	**55**	82	47	**2.90**	**0.76**	32% (**24–39**)	89% (**81–94**)	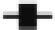	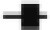
Devlin et al. (17)	Nox	Unclear	24	9	21	1	**82**	73	95	**3.20**	**0.06**	**96%** (**76–99**)	**70%** (**50–85**)	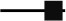	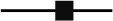
Dickson et al. (28)	Alpha, Pi GST	>60 ng/ml	7	2	**28**	**7**	**80**	78	80	7.50	**0.54**	50% (23–76)	93% (78–99)	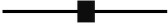	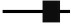
Feussner et al. (18)	SAA protein	1.5 mg/dL	8	1	24	4	**86**	89	86	**16.67**	**0.35**	67% (**34–90**)	96% (**79–99**)	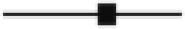	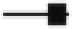
Hughes et al. (21)	Eosinophilia	Unclear	**27**	**10**	**25**	**9**	**73**	**73**	**74**	**2.62**	**0.35**	76% (**57–87**)	70% (**53–85**)	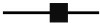	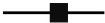
Kobayashi et al. (5)	GBP2 mRNA	20	**12**	**4**	**23**	**7**	**76**	**75**	**76**	**4.2**	**0.43**	63% (**38–83**)	85% (**66–95**)	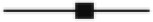	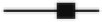
Kuse et al. (19)	Procalcitonin	5.9 ng/mL	11	7	22	0	**82**	**61**	**100**	**4.14**	**0**	100% (**71–100**)	**75%** (**56–90**)	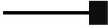	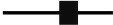
Lun et al. (22)	IL-2R	+631 IU/Ml	**56**	**5**	**45**	**13**	**85**	**92**	**78**	**7.3**	**0.2**	81% (**69–89**)	89% (**78–96**)	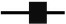	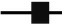
Massoud et al. (24)	C4	< 0.31 gm/L	**24**	**8**	**13**	**1**	**80**	74	94	**2.55**	**0.04**	97% (**79–99**)	62% (**38–81**)	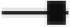	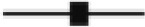
Molajoni et al. (30)	HLA class I antigen	>2.1 μg/mL	8	2	6	0	**88**	**80**	**100**	**4**	**0**	**100%** (**63–100**)	**75%** (**35–97**)	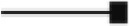	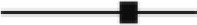
Nagral et al. (29)	Alpha GST	11.4 ng/mL	**24**	**11**	**7**	**14**	**55**	69	33	**1.03**	**0.95**	63% (**45–78**)	39% (**17–64**)	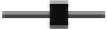	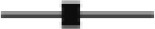
Okubo et al. (20)	CHMP2B	0.3330	**16**	**4**	**16**	**4**	**80**	80	80	**4**	**0.2**	80% (**56–94**)	80% (**56–94**)	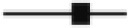	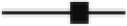
Rodriguez-Peralvares et al. (25)	Eosinophilia	0.46 × 10^9^/L	104	43	115	134	**55**	49	68	1.61	0.77	44%(39–48)	73% (65–80)	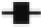	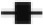
Schütz et al. (27)	GDCFDNA	>10%	**205**	**6**	**82**	**22**	**95**	**97**	**78**	**13.1**	**0.11**	90.3% (**85–93**)	93% (**86–97**)	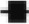	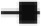
Wang et al. (26)	Eosinophilia	0.145 × 10^9^/L	**11**	**2**	**14**	**13**	**63**	85	52	**3.67**	**0.62**	46% (**25–67**)	88% (**62–98**)	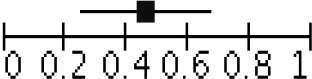	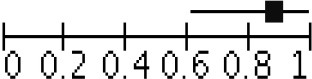

*Non-invasive markers were tested in patients who underwent liver transplantation to diagnose allograft rejection. Test parameters were derived from study records, whenever possible. In the case of missing parameters, data were calculated from contingency tables (Note: imputed data is marked in bold)*.

**Figure 2:**
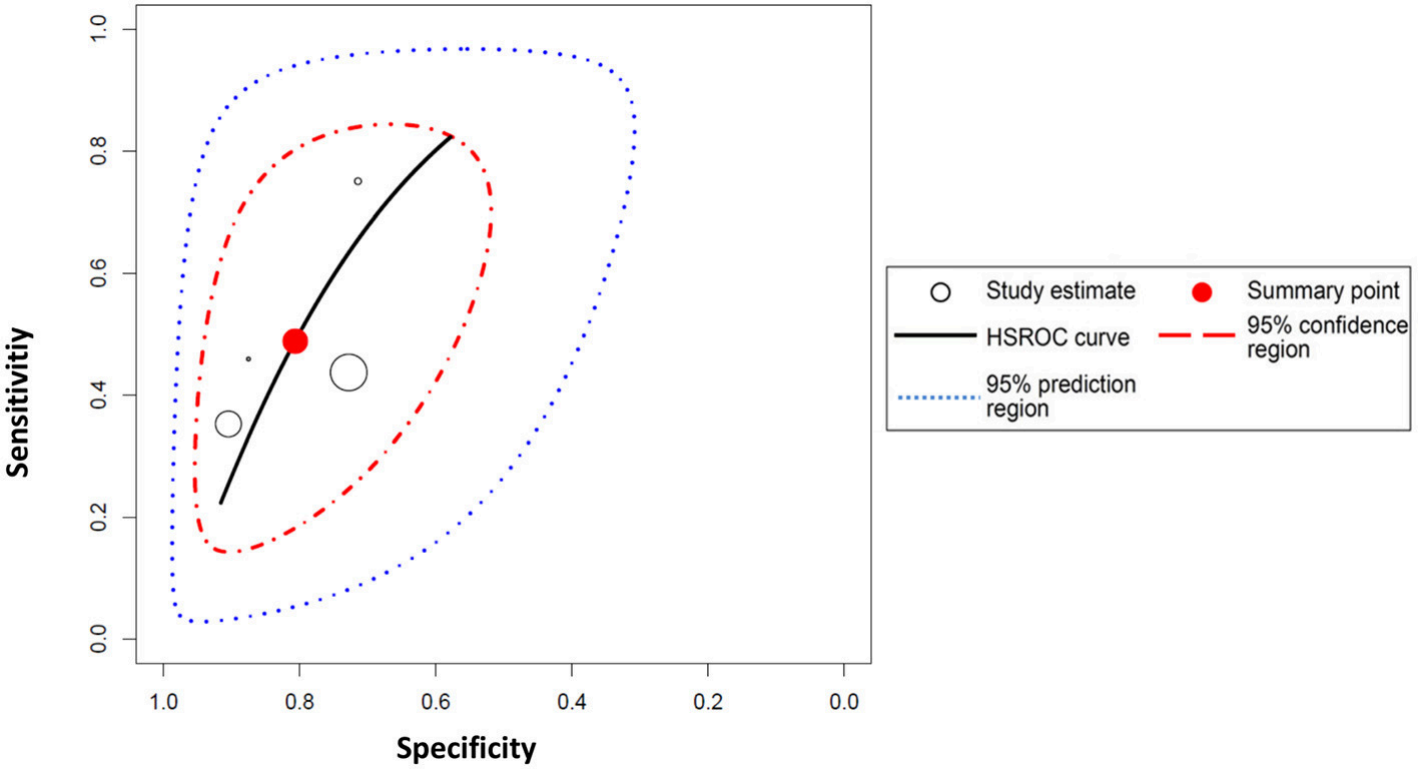
Hierarchical summary receiver operating characteristic (HSROC) curve for eosinophilia. The HSROC curve composed of studies examining the diagnostic value of eosinophilia in noninvasively diagnosing acute allograft rejection in patients undergoing liver transplantation is shown. Each individual study is represented by a circle; the blue line represents the confidence interval, and the red line represents the credibility interval.

**Figure 3:**
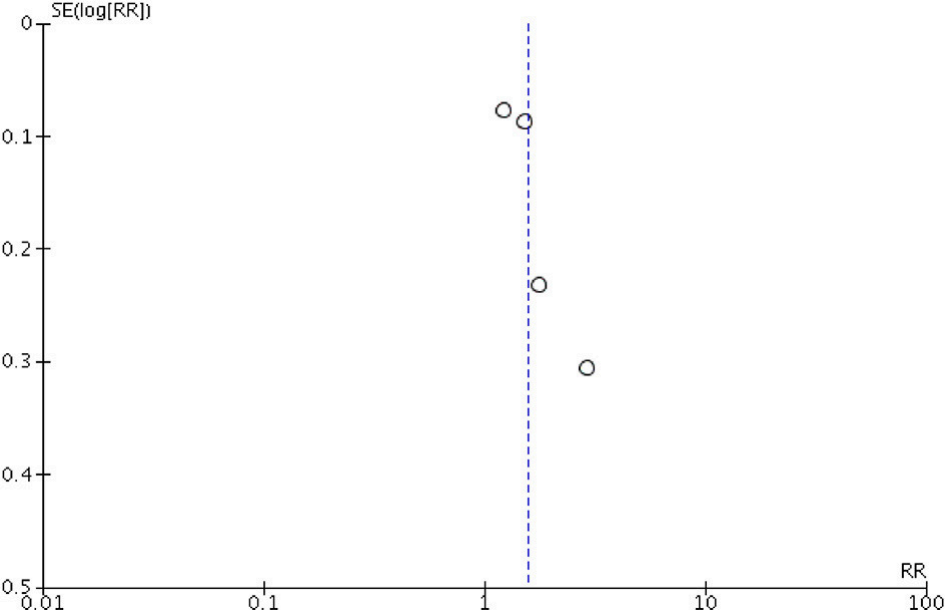
Funnel plot for the meta-analysis of studies testing eosinophilia as a noninvasive biomarker. Relative risk (RR) was plotted against the standard error (SE) of each study. The outcomes are represented by dots. Evidence of considerable bias is demonstrated.

Peripheral blood eosinophil count was analyzed in 4 studies that included 805 patients and 1,076 sample points (21, 23, 26) for meta-analysis. The studies were pooled in an HSROC to illustrate the diametrical sensitivity and specificity (Figure 2). Blood eosinophilia demonstrated a pooled sensitivity of 50% (95% CI: 0.18–0.78) and specificity of 80% (95% CI: 0.62–0.92). The DerSimonian–Laird random effect method was used to test the overall effect of peripheral blood eosinophilia on accurately diagnosing AR, and single study RRs were calculated: patients positive for eosinophilia had a 1.56 times higher risk (95% CI: 1.21–2.02) of AR compared with patients negative for eosinophilia (*p* = 0.0006). The results are graphically illustrated in a forest plot in Table 4. The heterogeneity among the studies was moderately high (72%, calculated by *I*^2^ statistics). Furthermore, RR and effect sizes were plotted against each other in a funnel plot to demonstrate possible asymmetry between the studies. The empty left side of the graphic shows existing bias, the origin of which may have been either publication bias, clinical heterogeneity or methodological heterogeneity (Figure 3).

**Table 4:** Meta-analysis for the test validity of peripheral blood eosinophilia in diagnosing acute allograft rejection.

	**Index test +**		**Index test −**				**Risk ratio (95% CI)**
**Study**	**TP**	**Total**	**FN**	**Total**	**Weight**	**Risk ratio (95% CI)**	
Hughes et al. (21)	27	37	9	36	0.13	2.92 (1.60–5.31)	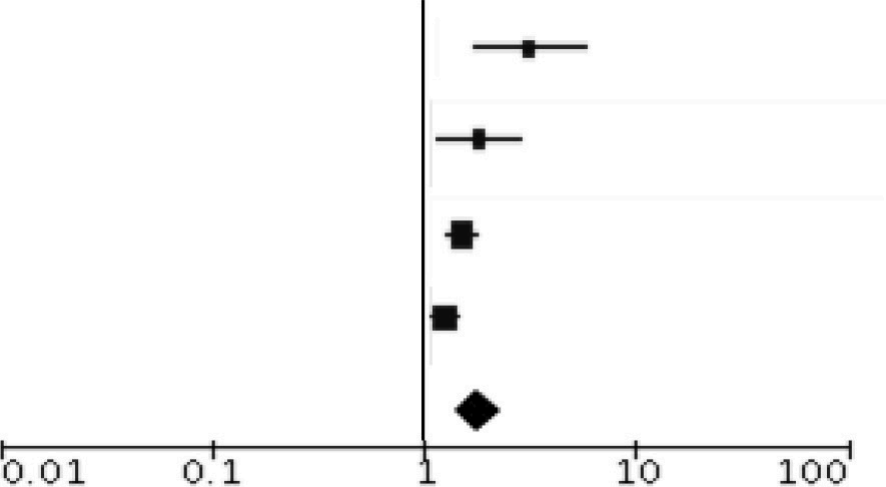
Wang et al. (26)	11	13	13	27	0.18	1.76 (1.12–2.77)	
Barnes et al. (23)	53	65	113	210	0.34	1.52 (1.28–1.80)	
Rodriguez-Peralvarez et al. (25)	104	147	134	231	0.36	1.22 (1.05–1.42)	
Total (95% CI)		262		504	1	1.56 (1.21–2.02)	

*Each study is shown by the point estimate of the risk ratio (RR) and the respective 95% confidence interval (CI), represented by the lines. The RR was calculated using the true positive (TP) value for blood eosinophilia and total number of eosinophilia-positive patients for the index test-positive group (TP/Total+) and the false positive (FP) value for blood eosinophilia and total number of eosinophilia-negative patients for the index test-negative group (FP/Total–). The combined RRs and CIs are represented by the diamond. The DerSimonian and Laird random effect model was used. I^2^ statistics was used as a measure of heterogeneity. A statistically significant overall effect was obtained (P = 0.0006). Heterogeneity: Tau^2^ = 0.04; Chi^2^ = 10.89; df = 3 (P = 0.01); I^2^ = 72%. Test for overall effect: Z = 3.44 (P = 0.0006)*.

The sample size varied among the studies that tested for eosinophilia and was not equally distributed. Rodriguez-Peralvarez et al. (25) included 615 patients (76%), Wang et al. (26) included 37 patients (5%), Barnes et al. (23) included 101 patients (13%), and Hughes et al. (21) included 51 patients (6%). Furthermore, the time points for the index test varied greatly. Wang et al. (26) studied blood eosinophilia as a marker of late AR and performed the index test and reference test later than 6 months following transplantation, whereas the other three studies (21, 23, 25) analyzed samples taken within days of transplantation.

#### Test Accuracy of Single Studies

Lun et al. (22) (69 AR cases, 119 samples) studied the accuracy of lymphocyte subset distribution, expression of T-cell activation markers and concentration of soluble interleukin-2 receptor (IL-2R) as markers of AR. There was upregulation of the IL-2R (CD25) on CD4+ and CD8+ T cells accompanied by an increase in soluble IL-2R in patients with AR. Indeed, soluble IL-2R exhibited the highest diagnostic efficiency on the day of rejection (+LR 14.49, -LR 0.44) and when measuring the difference between 3 days prior to and the day of rejection (+LR 7.3, -LR 0.2). The specificity and sensitivity of a threshold of >3,850 IU/ml on the day of AR was 96% (95% CI: 0.86–0.99) and 56% (95% CI: 0.28–0.62), respectively; the PPV and NPV were 83 and 85%, respectively. A threshold value of >631 IU/ml (the difference between 3 days prior to and the day of rejection) yielded the highest sensitivity and specificity values (81% [95% CI: 0.69–0.89] and 89% [95% CI: 0.78–0.96], respectively). The cut-off values were determined using the ROC curve. A multivariate analysis was performed to improve the diagnostic efficacy by a combination of best markers. However, IL-2R on day of biopsy, time course of IL-2R, and CD4 between 3 days before biopsy and the day of biopsy showed a correct classification of 71%, demonstrating no improvement over soluble IL-2R alone. Of note, the given and calculated PPV and NPV values differed. The risk of bias of the study was very low (Table 2).

Schütz et al. (27) (17 AR cases, 107 samples) studied graft-derived cell-free DNA in 107 patients undergoing LT due to various underlying liver diseases. Only samples taken ≤ 6 days before or 1 day after biopsy were considered for analysis. Graft-derived cell-free DNA was obtained in stable patients, HCV+ patients and patients suffering from AR. The percentage was significantly elevated (29.6% [95% CI: 0.23–0.41]) compared with HCV+ patients (5.9% [95% CI: 0.04–0.11]) and stable patients (3.3% [95% CI: 0.03–0.037%]; *p* < 0.001). Moreover, the authors compared the diagnostic ability of graft-derived cell-free DNA to that of normal liver function testing and demonstrated independent information on graft integrity. Graft-derived cell-free DNA with a threshold of 10% yielded a sensitivity of 90.3 (95% CI: 0.74–0.98) and specificity of 92.9% (95% CI: 0.89–0.95). However, the study was high in bias due to unclear exclusion criteria (13 patients were excluded without reasonable explanations; Table 2). Moreover, they did not include HCV+ patients with stable patients when analyzing for sensitivity and specificity. Hence, that led to a significant decline in test accuracy and increase in the threshold when they compared HCV+ patients to patients with biopsy-proven AR (sensitivity 75% [95% CI: 0.55–0.89]; specificity 84.2% [95% CI: 0.71–0.92]).

Feussner et al. (18) (14 AR cases, 42 samples) studied serum amyloid A protein (SAA) and found a high positive LR (16.67). The sample size of the study was small and demonstrated a sensitivity of 66.7% (95% CI: 0.35–0.9) and specificity of 96% (95% CI: 0.97–1). There was no pre-specified threshold, and a cutoff point of 17 mg/dl was derived from the ROC curve. Interestingly, no statistical correlation was found between SAA and CRP (*n* = 37; *r* = 0.237; *p* = 0.157).

Dickson et al. (28) (14 AR cases, 44 samples) studied alpha-glutathione S-transferase (alpha-GST) and pi-glutathione S-transferase (pi-GST) as potential markers of hepatocyte and biliary epithelial cell injury, which are considered possible indicators of AR. Alpha-GST was found to have a positive LR of 7.5 and a negative LR of 0.54, with a sensitivity of 50% (95% CI: 0.23–0.76) and specificity of 93% (95% CI: 0.78–0.99). Patients with no and mild rejection were grouped and compared with those with moderate and severe rejection; however, mean values of alpha-GST and pi-GST were indistinguishable between the two groups (data not shown in the manuscript).

Barnes et al. (23) (166 AR cases, 275 samples), Rodriguez-Peralvarez et al. (25) (238 AR cases, 690 samples), Wang et al. (26) (24 AR cases, 40 samples), and Hughes et al. (21) (36 AR cases, 71 samples) all studied the peripheral blood eosinophil count as a noninvasive marker of AR. Three of them (21, 23, 25, 26) had very low bias (Table 3). Wang et al. had the highest positive LR (3.67), followed by Barnes et al. and Hughes et al. Barnes et al. demonstrated the highest specificity (% [95% CI: 0.81–0.94]) at an absolute eosinophil count (AEC) cut-off value of 0.145 × 10^9^, but reported a very low sensitivity (32% [95% CI: 0.24–0.39]). Hughes et al. demonstrated a high NPV (94%) and the highest sensitivity (76% [95% CI: 0.57–0.87]); however, the pre-specified threshold led to a high risk of bias. Wang et al. also stated that an AEC of 0.145 × 10^9^ and a relative eosinophil count (REC) of 2.3% demonstrated the highest Youden index, with areas under the ROC curves of 0.746 and 0.813, respectively. Furthermore, an REC of >2.3% was able to predict late AR (time point >6 months after LT) with a specificity of 87.5% and a sensitivity of 75%. Although REC shows higher accuracy parameters, the AEC was used in Table 3 to preserve unanimity within the studies of eosinophilia. Furthermore, Barnes et al. and Rodriguez-Peralvarez et al. studied the accuracy of the AEC vs. the change in eosinophil count in predicting the improvement or deterioration of the histological grade of rejection as a response to treatment in a subgroup of patients who underwent a second biopsy (*n* = 45 and *n* = 89, respectively). Barnes et al. found no significant difference in the AEC or the REC among those patients who subsequently improved, remained stable or deteriorated histopathologically following corticosteroid therapy; whereas, Rodriguez-Peralvarez et al. discovered that the change in AEC between the first and second biopsies was closely correlated with the histological course of AR (AUC 0.72 [95% CI: 0.66–0.78]). Of note, the data from Hughes et al. and Rodriguez et al. showed disagreement between the stated and extracted PPVs and NPVs by conventional contingency table (21, 25).

Devlin et al. (17) (33 AR cases, 55 samples) studied plasma concentrations of the acid-labile nitroso-compound (NOx) as a possible marker (+LR 3.20; –LR 0.06). They demonstrated that NOx increased during AR (*p* < 0.0001) in association with histopathological grading and decreased after administration of glucocorticoids. It is unclear from the manuscript whether a threshold was predetermined for NOx, which would interfere with the reliability of the test accuracy. In addition, the author studied the relationship between NOx and circulating TNF-alpha and IL-2R, with a predetermined threshold for circulating TNF-alpha.

Studies of Okubo et al. (20) (20 AR cases, 80 samples) and Massoud et al. (24) (33 AR cases, 62 samples) were similarly designed. The authors used proteomics and ELISA to test blood samples to discover possible markers of AR. In both studies, the discovery set was composed of patients undergoing LT due to hepatitis C infection (with or without histopathological signs of AR). Note, Okubo et al. also included patients with liver dysfunction and healthy volunteers without any signs of AR. Next, a completely separate group of patients was set up for validation (ELISA) of markers that were revealed in the discovery set. The validation panel were still sub-grouped into patients with AR and patients without AR after LT. Massoud et al. identified 41 proteins, while C4 and C1q were both independent predictors for AR with sensitivities of 97% (95% CI: 0.79–0.99) and 56% (95% CI: 0.35–0.76) and specificities of 62% (95% CI: 0.38–0.81) and 86% (95% CI: 0.64–0.97), respectively. A noteworthy secondary outcome was the increased marker specificity of 81% and sensitivity 96% when C4 (cut-off < 0.31 gm/L) was combined with ALT (cut-off > 70 IU/ml). Taken together, C4 demonstrated the best test accuracy in differentiating patients with and without AR. Okubo et al. performed microarray analysis and identified 57 autoantibodies that were upregulated in the AR group; he then selected four autoantibodies (multivesicular body protein 2B [CHMP2B], KCTD14, voltage-gated subfamily A regulatory beta subunit 3 [KCNAB3] and triosephosphate isomerase 1 [TPI1]) by fold change and antibody intensity. KCNAB3 was found to be significantly higher in the AR group compared with the group with liver dysfunction and no AR (+LR 2.25; –LR 0.17). CHMP2B and TPI1 were both significantly overexpressed in the AR group compared with the other control groups. The levels of these antibodies increased only around the time of acute cellular rejection, making them good candidates for diagnostic molecular markers. CHMP2B showed outstanding performance, with an AUC of 0.86 (95% CI: 0.75–0.97) and 80% specificity and 80% sensitivity at a cut-off value of 0.33.

Kuse et al. (19) (10 AR cases, 40 samples) tested whether procalcitonin (PCT) allowed differentiation between infection and AR in cases of fever following LT. The authors demonstrated that PCT had a high predictive value in differentiating between AR and infection, with an AUC of 0.93. The highest sensitivity and specificity values were found at a cut-off of 5.9 ng/ml, with 100% sensitivity (95% CI: 0.71–1) and 75% specificity (95% CI: 0.56–0.9). Renna Molajoni et al. (30) (8 AR cases, 16 samples) studied whether serum HLA class I antigen was associated with AR: a cut-off value of >2.1 g/ml yielded a sensitivity of 100% (95% CI: 0.63–1) and a specificity of 75% (95% CI: 0.35–0.97). However, both studies had low reliability due to underpowered sample sizes and restricted inclusion criteria.

Nagral et al. (29) (38 AR cases, 56 samples) evaluated the efficacy of alpha-GST as a marker of AR in comparison with standard liver function tests (ALT and bilirubin). At a cut-off value of >11.4, alpha-GST demonstrated a sensitivity of 63.1% (95% CI: 0.45–0.78) and a specificity of 38.8% (95% CI: 0.17–0.64); whereas, ALT (cut-off >40 IU/ml) yielded a sensitivity of 97.4% and specificity of 16.6%. Alpha-GST was also tested as a marker of successful anti-rejection therapy in 16 patients. However, alpha-GST only decreased in 7 of 10 cases following anti-rejection therapy (*p* = 0.9) and, thus, was not linked to histological improvement.

Kobayashi et al. (5) (19 AR cases, 46 samples) analyzed the diagnostic efficacy of guanylate-binding protein 2 mRNA (GBP2) and interferon regulatory factor 1 mRNA (IRF1) as markers of AR using real-time PCR. Patients with liver dysfunction (LD) were included and further subgrouped into LD with AR vs. LD without AR patients. Although both IRF1 and GBP2 were higher in patients with LD (independent of AR) than in controls, only GBP2 was higher in LD with AR patients compared to LD without AR patients (+LR 4.2; –LR 0.43). A cut-off value of 20 produced a sensitivity and specificity of 63% (95% CI: 0.38–0.83) and 85% (95% CI: 0.66–0.95), respectively. A noteworthy result was that GBP2 was unable to distinguish between AR and HCV recurrence (*p* = 0.2). The study was high in bias for patient selection because of inappropriate patient exclusion (subjective exclusion criteria, e.g., “rejection, infection, or recurrence of primary disease”), which may have led to an overestimation of study findings (Table 2).

## Discussion

The present systematic review and meta-analysis has summarized the current status of noninvasive diagnostic biomarkers to diagnose AR following LT and, in doing so, has identified the best-evaluated diagnostic parameters. In total, 10 blood markers were identified to diagnose AR, while the AEC was validated by most studies and soluble IL-2R exhibited superior study results. In the following paragraphs, we will discuss the most important challenges currently limiting the establishment of a noninvasive diagnostic markers in the diagnosis of AR after liver transplantation.

A summary of the study exclusion criteria can be found in Supplementary Figure 1. When we initially designed the present systematic review, we did not anticipate the number of studies that would not consider test accuracy (e.g., sensitivity and specificity) or timely connect the reference test to the index test (16% and 8%, respectively). Test accuracy is essential in assuring study comparability and performance. The timely correlation between the reference test and the index test is also important as anti-rejection therapy should be initiated on the same day as diagnosis. Therefore, appropriate methods and statistical tests remain of utmost importance for the translation of findings into routine clinical practice. However, when we reviewed the literature, more than 95% of all screened studies did not consider these two fundamental characteristics.

It appears plausible that among the 10 identified markers, IL-2R expression in peripheral blood leukocytes, with a sensitivity, specificity, NPV and PPV all above 80% and a considerable sample size (119 patients), demonstrated the most promising diagnostic accuracy for translation into clinical use. This was underscored by the trial set up, patient criteria and very low risk of bias. Although it did not show as strong a diagnostic accuracy, peripheral blood eosinophilia was studied by Hughes et al. (21), Wang et al. (26), Rodriguez-Peralvarez et al. (25), and Barnes et al. (23), who included considerable sample sizes and consequently had high reliability of their results. Rodriguez-Peralvarez et al. demonstrated a significant correlation between AEC and histological improvement in response to anti-rejection therapy (25), whereas Barnes et al. found no statistically significant change in AEC in response to treatment (23). The discrepancy in study results for the utility of peripheral blood eosinophilia in accurately differentiating between different grades of rejection and for predicting histopathological improvement after appropriate anti-rejection therapy may be due to the considerable difference between the two studies' sample sizes, 487 vs. 45. Another contributing factor may be the sample grouping into no–mild rejection and moderate–severe rejection groups, which could have led to bias. In conclusion, peripheral blood eosinophilia was the most frequently tested marker, but its clinical use as a single marker of AR is not supported due to low data accuracy. However, eosinophilia might be useful as a complementary test to indicate the need for closer noninvasive monitoring of transplant patients for possible liver biopsy.

Schütz et al. (27) studied graft-derived cell-free DNA in the largest cohort study but also excluded patients for undefined reasons. Test accuracy was high, although HCV+ patients were excluded from stable patients in the sensitivity and specificity analyses. Indeed, test accuracy decreased significantly when HCV+ patients were included, yielding a sensitivity of 75% and specificity of 84.2%. Taken together, the concept of graft-derived cell-free DNA is promising, although the study design was imprecise.

In a few studies, true positive, false positive, true negative and false negative values were used to calculate diagnostic accuracy, and the sensitivity, specificity, PPV, NPV, LR, and RR were either not stated at all or only partially stated. Furthermore, Hughes et al. and Rodriguez et al. showed inconsistency in the stated and derived PPV and NPV values, raising concerns about the reliability of the reported AEC data (21, 25). Therefore, the statistical error obviously increased as we constructed contingency tables for our own calculations with missing accuracy values. Indeed, calculated sensitivity, specificity, PPV and NPV values did not always match the stated values (21, 22, 25).

Six studies (19, 22, 23, 25, 26, 29) were assessed as having low risk of bias, while nine studies (5, 17, 18, 20, 21, 24, 27, 28, 30) were found to have high risk of bias in all tested categories. Study results could have been affected by inappropriate patient exclusion strategies, previously defined threshold values, inappropriate patient flow and timing.

No pediatric study (*n* = 27) did match our inclusion criteria. Most reviewed studies do not include adults and children at the same time, wherefore a possible particular finding will not be applicable to both, the young and the old transplant recipients. There are differences of the incidence of acute rejection with increasing age (31). In addition, there are changes in immune response with age, why some e.g. cytokines are low in young age, but high in old age (31). Furthermore, immunosuppressive regime is more complex in pediatric patients with longer expected period of intense immunosuppression and changes in pharmacokinetics in older patients (32). Therefore, we did not consider pediatric patients in our systematic review.

In addition to the study design, another important factor currently limiting the success of recent and past studies is the lack of a robust endpoint that plays a crucial role in objectively assessing the diagnostic efficacy of a particular biomarker. Liver biopsy is the gold standard for the diagnosis of acute rejection and the Banff Working Group on Liver Allograft Pathology has defined histopathological finding of AR (33). Nevertheless, percutaneous biopsy and diagnosis of AR is characterized by a lack of reproducibility between experienced and inexperienced pathologists (34, 35). Most diagnostic biomarkers consist of proteins, which are elevated not only in AR, but also during inflammation and infectious diseases, thus greatly lacking in specificity (e.g., IL-2). Bacterial, viral and fungal infections are one of the most common and challenging complications following LT. While the immunosuppressive regimens do not vary substantially between different solid organ transplantations, similar infection patterns and pathogens can be encountered (36). The postoperative course of liver transplant patients is often complicated by various infections like reinfection with hepatitis B and C viruses. In addition, every infection can timely overlap with AR episodes, rendering the differential diagnoses and treatment difficult. Most studies do not consider this particular problem to differentiate between AR from infection. For instance, AR and recurrent hepatitis C often coexist at the same time (37). A multicenter study performed by Regev et al. tested the reliability of histopathologic findings between recurrent HCV infection and AR (38). The colleagues unraveled low interobserver and intraobserver agreement rates, with a kappa score of smaller than 0.4. The same diagnostic uncertainty exists with recurrence of autoimmune hepatitis (AIH) and the occurrence of *de novo* autoimmune hepatitis (dnAIH) after liver transplantation, which can both lead to graft dysfunction if not timely treated. Interestingly, history HCV infection and interferon gamma therapy are both related to dnAIH occurrence post-transplantation (39). Moreover, study of the inflammatory infiltrates in the livers of transplant pediatric patients showed that antibodies against T-bet (transcription factor of T helper cells 1) was lower in dn-AIH than in the AR and AIH group (40). Risk of recurrence of AIH post-transplantation is related to risk factors such as HLA-DR3 or HLA-DR4 positivity (41) and early withdrawal of corticosteroids (42, 43). Taken together, autoimmunity and infection can change histological findings and make it difficult to diagnose AR by histology. This is analogue to an ideal biomarker that must not only diagnose the AR, but also be able to correctly differentiate to existing inflammatory states. Note, failure to differentiate between infection, recurrent HCV and AR may result fatal cumbersome by immunosuppression that orchestrate immune response and perifocal immunological changes (44, 45).

A stratification analysis was considered in order to examine the studies in separate sample size layers to test for heterogeneity. However, while only 4 studies were considered for the meta-analysis, stratification would lead to low number with reduced subgroups. Since the estimation of the degree of heterogeneity in a model with random effects depends to a large extent on the number of studies, a stratification means smaller subsets with worse estimates of the amount of heterogeneity (46). The sample sizes of the studies included in the meta-analysis are: 71, 40, 275, 690. Hence, a stratification in two or more groups cannot be made meaningfully. Therefore, we decided to quantify the heterogeneity between the studies using the random-effect model described by DerSimonian and Laird (47).

## Conclusion

The present review and meta-analysis systematically overviews research on the use of noninvasive markers to diagnose AR after LT. Interestingly, although only tested in one study, IL-2R exhibited superior sensitivity/specificity underscored by a decent sample size and low bias for all screened parameters. Nevertheless, liver biopsy remains superior to the noninvasive approach of diagnostic biomarkers as most of the marker study designs were inferior, hindering the possible translation of this noninvasive technique into routine clinical use at this time. This is complicated by the fact, that a robust endpoint for AR is missing and the evaluation of histopathological findings of AR are influenced by intra- and interobserver variability and occurring infections. Therefore, an appropriate clinical trial design for validating the diagnostic accuracy of a potential noninvasive marker for diagnosing AR remain indispensable.

## Author Contributions

FK and EK elaborated hypothesis, constructed the search algorithm and performed the literature search systematically. FK, EK, MS, and AL wrote the manuscript. SG and KK substantial contributed to the acquisition of a large part of the extracted data. IS and KS critically revised the manuscript and interpreted the data. EK and KS performed the statistical analysis. JP and LF added important intellectual content to the manuscript. FK, EK, MS and AL edited the revision of the manuscript. All authors read and approved the final version of the manuscript.

### Conflict of Interest Statement

The authors declare that the research was conducted in the absence of any commercial or financial relationships that could be construed as a potential conflict of interest.

## Supplementary Material

Supplementary Figure 1 Overview of the reasons certain studies were not included in the meta-analysis. Of the initial 560 references retrieved from the databases, 104 were filtered for full-text paper review after title and abstract screening. Of these, 15 studies fulfilled all inclusion criteria and were included in the systematic review. Note, conference papers are not depicted in this graph.

Supplementary Figure 2 Overview of risk of bias and applicability assessment of diagnostic studies. The Quality Assessment of Diagnostic Accuracy Tool (QUADAS-2) (12) was used to evaluate concerns regarding the risk of bias and applicability of study findings. The signaling questions were answered according to the judgment of two independent reviewers. Concerns regarding study applicability were low for all studies (>90%), while risk of bias varied for different components. Overall, the highest bias was found for patient flow and timing, and the lowest bias was for the index test.

Supplementary Table 1 Items relevant to the Quality Assessment of Diagnostic Accuracy Studies tool QUADAS-2 protocol.
