# Supplementary figures and images for: Diagnostic Biomarkers to Diagnose Acute Allograft Rejection After Liver Transplantation: Systematic Review and Meta-Analysis of Diagnostic Accuracy Studies
Source: Front Immunol. 2019 Apr 11;10:758. doi: 10.3389/fimmu.2019.00758 (PMC6470197; doi:10.3389/fimmu.2019.00758)

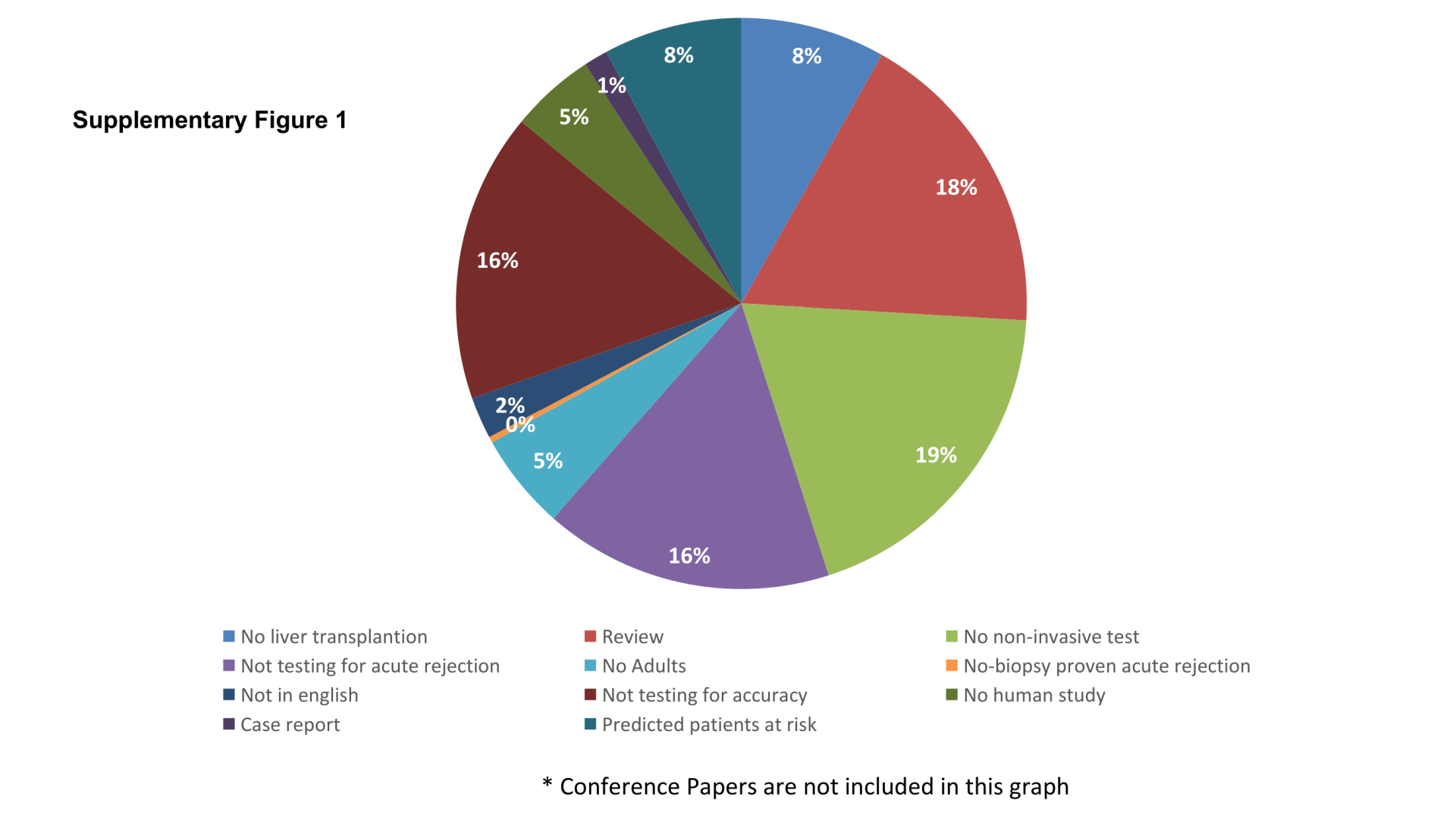

Supplement: Supplementary Figure 1 — Overview of the reasons certain studies were not included in the meta-analysis. Of the initial 560 references retrieved from the databases, 104 were filtered for full-text paper review after title and abstract screening. Of these, 15 studies fulfilled all inclusion criteria and were included in the systematic review. Note, conference papers are not depicted in this graph. [file Image_1.tif]

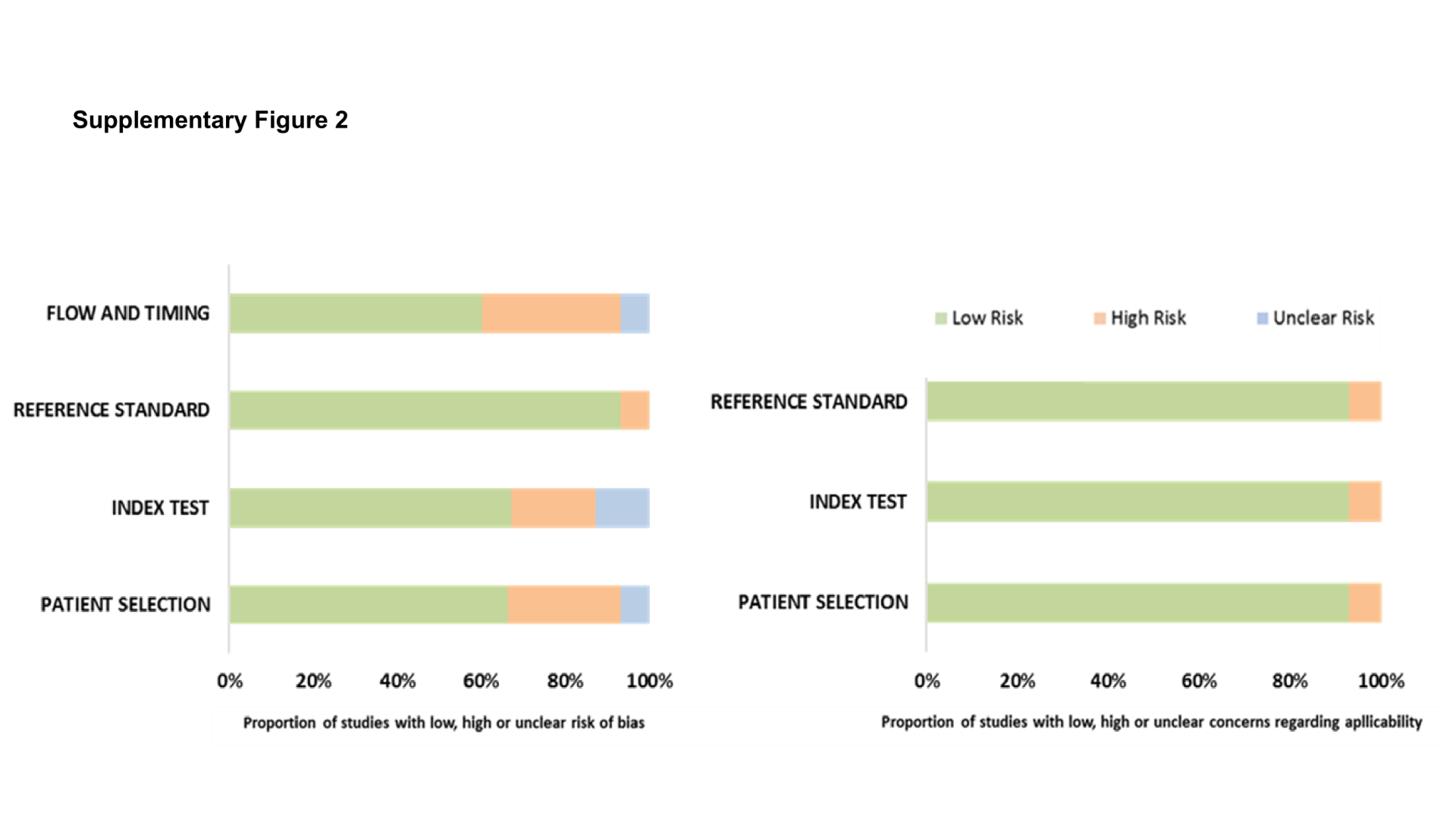

Supplement: Supplementary Figure 2 — Overview of risk of bias and applicability assessment of diagnostic studies. The Quality Assessment of Diagnostic Accuracy Tool (QUADAS-2) (12) was used to evaluate concerns regarding the risk of bias and applicability of study findings. The signaling questions were answered according to the judgment of two independent reviewers. Concerns regarding study applicability were low for all studies (>90%), while risk of bias varied for different components. Overall, the highest bias was found for patient flow and timing, and the lowest bias was for the index test. [file Image_2.tif]
