# Supplementary material for: Diagnostic Biomarkers to Diagnose Acute Allograft Rejection After Liver Transplantation: Systematic Review and Meta-Analysis of Diagnostic Accuracy Studies
Source: Front Immunol. 2019 Apr 11;10:758. doi: 10.3389/fimmu.2019.00758 (PMC6470197; doi:10.3389/fimmu.2019.00758)
Supplement: Supplementary Table 1 — Items relevant to the Quality Assessment of Diagnostic Accuracy Studies tool QUADAS-2 protocol. [file Table_1.docx]

| **Domain 1:** Patient selection | Was a consecutive or random sample of patients enrolled? |
| --- | --- |
|  | Was a case-control design avoided? |
|  | Did the study avoid inappropriate exclusions? |
|  | **Could the selection of patients have introduced bias?** |
|  | **Is there concern that the included patients do not match the review question?** |
| **Domain 2:** Index test | Were the index test results interpreted without knowledge of the results of the reference standard? |
|  | If a threshold was used, was it pre-specified? |
|  | **Could the conduct or interpretation of the index test have introduced bias?** |
|  | **Is there concern that the index test, its conduct, or interpretation differ from the review question?** |
| **Domain 3:** Reference standard | Is the reference standard likely to correctly classify the target condition? |
|  | Were the reference standard results interpreted without knowledge of the results of the index test? |
|  | **Could the reference standard, its conduct, or its interpretation have introduced bias?** |
|  | **Is there concern that the target condition as defined by the reference standard does not match the review question?** |
| **Domain 4:** Flow and timing | Was there an appropriate interval between index test(s) and reference standard? |
|  | Did all patients receive a reference standard? |
|  | Did patients receive the same reference standard? |
|  | Were all patients included in the analysis? |
|  | **Could the patient flow have introduced bias?** |

**Supplementary Table 1**: Items relevant to the Quality Assessment of Diagnostic Accuracy Studies tool QUADAS-2 protocol
